# Measures of heart rate variability in women following a meditation technique

**DOI:** 10.4103/0973-6131.66772

**Published:** 2010

**Authors:** Hyorim An, Ravi Kulkarni, R Nagarathna, HR Nagendra

**Affiliations:** Vivekananda Yoga Research Foundation, Bangalore, India

**Keywords:** Heart rate variability, meditation, sampling entropy, pNN50, pNN30

## Abstract

Certain time domain, frequency domain and a nonlinear measure of heart rate variability are studied in women following a meditative practice called cyclic meditation. The nonlinear measure studied is the sampling entropy. We show that there is an increase in the sampling entropy in the meditative group as compared to the control group. The time domain measure called pNNx is shown to be useful in distinguishing between the meditative state and a normal resting state.

## INTRODUCTION

Cyclic meditation (CM) is a practice that originated in Prashanti Kuteeram, a yoga therapy center in South India. Briefly described, the practice consists of very slow movements performed with awareness of subtle shifts of balance and muscle tension during the entire exercise. People who go through the practice uniformly report a feeling of calmness and well-being at the end of the procedure. This paper describes the changes in certain measures of heart rate variability in women following the practice. The practice, performed with closed eyes, lasts about 25 min in the version used for this study. The effects of this practice on heart rate variability have previously been studied in healthy men.[1] That study examined the Fourier power spectrum of the RR interval series and found a significant decrease in the low frequency (LF) component and a significant increase in the high frequency (HF) component following the practice, indicating a shift toward parasympathetic dominance.

There have been other studies on meditation and heart rate variability. Kubota *et al*.,[2] studied the relationship between cardiac autonomic function and activity of the medial frontal neural circuitry. Murata *et al*.[3] also observed an increase in the HF power and decrease in the LF/HF ratio. It has been noted in the literature that heart rate dynamics may be different between men and women.[3,4] These studies have reported increased complexity in approximate entropy and a higher HF component in short-term electrocardiogram recordings in women compared with men.[5] The aim of this study therefore was to examine certain time domain and frequency domain measures for women following the practice of CM. We also report changes in the sampling entropy (SampEn)[6] discussed below as this is a non-linear measure that has been found to be useful even for short-term recordings and has been widely discussed in the literature. The changes in SampEn and the pNNx measures that are described below have not been studied before in the context of heart rate variability. These measures are described in greater detail below.

## MATERIALS AND METHODS

### Subjects and study protocol

The subjects, 28 in number, were healthy female volunteers, resident at the center where this study was carried out. Their mean age was 25 ± 3.4 years. Each subject participated in two different sessions – one of CM lasting 23 min and another of supine rest (SR) lasting an equal number of minutes. Each session was on a different day. In the SR position, the subjects were asked to lie down and were asked to remain passive but awake with natural breathing. CM is a guided relaxation practice and the practitioner is supposed to allow the breath to flow naturally – there is definitely no attempt at controlling the breathing rate or trying to synchronize it with the body movements that are very slow. All subjects were given practice in the technique prior to the actual recording, which was performed in individual sessions with the subject following taped instructions.

The RR intervals were recorded for a period of 5 min before and after the practice of CM and the same protocol was also followed for the SR sessions. The data therefore consisted of 5-min recordings before and after each practice (CM and SR) for each subject. The RR intervals were recorded using RMS Polyrite D, a digitized polygraph manufactured by Recorders and Medicare Systems, Chandigarh, India. The sampling rate was 256 Hz and was interpolated at 4 Hz. Each recording was edited manually to detect and eliminate spurious noise.

### Frequency domain analysis

Fourier analysis of the RR-interval series was performed using the freely available HRV Analysis software version 1.1, developed by the Biomedical Signal Analysis Group at the University of Kuopio, Finland.[7] The 5-min RR interval recordings used for frequency domain analysis were detrended using smoothness priors and interpolated at 4 Hz. The absolute power and the power in normalized units in the LF band (0.04–0.15 Hz) and in the HF band (0.15–0.4 Hz) were used for analysis.

### Time domain analysis

In addition to the mean and the standard deviation of the RR intervals, measures derived from interval differences were also calculated. These were the root mean square of successive differences (RMSSD) and the pNNx measures. The earliest of the pNNx measures described in the literature was pNN50, which is the percentage of RR intervals that differ by more than 50 ms from its immediate successor.[8] A generalization of the pNN50 statistic, called the pNNx measures, was introduced soon after,[9] where × is an interval <50 ms. The aim of these added measures was to provide more robust discrimination between the groups than the pNN50 statistic and was applied successfully, e.g., to distinguish between healthy subjects while asleep and healthy subjects while awake. This study reports the pNN50 and the pNN30 statistic before and after each of the two sessions. The RMSSD, the square root of the mean squared differences of successive RR intervals, is another measure that is sensitive to HF (short-term) variations in the heart rate. Its changes are also reported here.

### Non-linear analysis

SampEn[6,10] is a non-linear measure that is used to quantify the amount of complexity in a time series and has been widely studied in the context of the RR-interval series. SampEn is a modified version of an earlier measure called approximate entropy (ApEn),[11] which has also been widely studied. SampEn quantifies the repetition of patterns in the series of RR intervals. Larger values of entropy correspond to greater complexity while smaller values imply more recognizable patterns in the data. These entropy measures, unlike most non-linear measures of heart rate variability, can be used on data taken for short periods. They depend on a parameter *m* (the length of the sequence being matched) and a recording of 10*^m^* – 20*^m^* RR intervals is judged sufficient to estimate the entropy.[12] SampEn was calculated for 300 RR intervals in this study, with *m*=2 and a tolerance of 0.2, using the program available at the PhysioNet website.[13]

## RESULTS

In describing the results below, the two practices of cyclic meditation and supine rest are abbreviated to CM and SR. The sessions before and after are referred to as PRE and POST.

### Frequency domain

Table 1 shows the means of the normalized LF and HF values and also the LF/HF ratio. Table 1 shows the actual power in both the LF (0.04–0.15 Hz) and the HF (0.15–0.4 Hz) bands. The data for the LF- and HF-normalized values were found to be normally distributed while the LF/HF ratios were not.

**Table 1:** Means and standard deviations

	CM pre	CM post	SR pre	SR post
LF	37.62 ± 12.53	31.61 ± 13.19	37.25 ± 19.75	38.80 ± 15.28
HF	62.38 ± 12.53	68.39 ± 13.19	62.75 ± 19.75	61.20 ± 15.28
LF/HF	0.69 ± 0.46	0.52 ± 0.37	0.81 ± 0.76	0.78 ± 0.67
LF (power)	334.69 ± 224.35	369.50 ± 348.61	364.88 ± 341.04	416.27 ± 266.46
HF (power)	691.19 ± 575.53	881.00 ± 722.31	744.81 ± 848.31	723.77 ± 512.41
pNN50	28.01 ± 19.66	34.16 ± 20.78	29.67 ± 20.71	30.69 ± 20.31
pNN30	49.92 ± 21.26	57.19 ± 16.50	50.17 ± 22.55	53.27 ± 18.56
RMSSD	52.10 ± 24.50	58.21 ± 26.36	51.07 ± 26.78	55.50 ± 25.84
SampEn	2.135 ± 0.367	2.218 ± 0.470	2.197 ± 0.434	2.191 ± 0.283
HR	72.29 ± 8.43	68.43 ± 7.73	72.00 ± 8.72	70.16 ± 8.15

LF = Low frequency, HF = High frequency, RMSSD = Root mean square of successive differences, SampEn = Sampling entropy, HR = Heart rate

Paired sample *t*-tests for the normalized LF data showed the following: the difference between the means of the two PRE sessions was not significant. The means in the POST session were significantly different (*P*=0.007), with the CM group showing a lower value [Table 1]. The CM group showed a significant decrease (*P*=0.011) in LF while the SR group did not show any significant change. Because this analysis was on the normalized units, the *P* values for the HF data are obviously exactly the same, with all differences reversed in sign. Thus, the CM group showed a significant increase (*P*=0.011) in HF.

The Wilcoxon test for the LF/HF ratio showed the following: there is a significant reduction for the CM group (*P*=0.011) while the reduction for the SR group is not significant. The PRE values of the two groups are not significantly different while the POST values are (*P*=0.007), with the CM group showing a lower value [Table 1].

The absolute powers were not normally distributed and the HF absolute power showed a significant increase (*P*=0.034, Wilcoxon test) between the PRE and POST CM sessions. There were no significant changes or differences in the absolute power otherwise.

### Time domain

The heart rate data were normally distributed and the mean decreased significantly after both practices Table 1 (*t*-tests, *P*<0.001 after CM and *P*=0.01 after SR). The difference between the two PRE sessions was not significant while the heart rate after CM was significantly less than that after SR (*P*=0.024).

The means and standard deviations of pNN50 and pNN30 are given in Table 1. The data were normally distributed. *t*-tests show that the pNN50 count increased significantly after CM (*P*=0.007). No other significant changes/differences were observed in the pNN50 statistic. The pNN30 statistic shows a significant increase after CM (*P*=0.003) and also shows a significant difference between the POST CM and SR sessions (*P*=0.024), the PRE difference not being significant.

The RMSSD data were normally distributed. Their behavior mimics that of the pNN50 statistic; the RMSSD increased significantly after the CM session (*P*=0.037) and no other changes/differences were observed. Table 1 shows the means and standard deviations.

### SampEn

The data were normally distributed. An increase in the CM session of SampEn (2, 0.2) was seen, but this was not significant (*P*=0.268). A slight decrease was observed in the SR session, which was not significant (*P*=0.939). There were no significant differences between the sessions. Table 1 shows the means and the standard deviations.

## DISCUSSION

This study was performed with the aim of looking at changes in certain measures of heart rate variability after the practice of CM in women.

The observed decrease in the LF power (normalized units) and the corresponding increase in the HF power are similar to that observed in men after the practice of CM, and Patil[1] suggests that the practice results in a shift toward vagal dominance. The observed decrease in the heart rate is also clearly an indication of parasympathetic dominance after the practice. Telles, Reddy and Nagendra[14] have shown that this reduction in the heart rate after the practice is coupled with a reduction in the respiratory rate and oxygen consumption.

Mietus *et al*.[15] carried out three different comparisons on data from the PhysioNet databases and demonstrated that separation between groups based on their RR intervals is consistently improved by using threshold values <50 ms; the threshold values being as low as 10 ms. Thus, the pNNx measures with × <50 provide useful information about the very short-term control of sinus rhythm dynamics. In the data discussed here, this is clearly demonstrated by the fact that the pNN50 measure could not distinguish between the CM and the SR sessions while the pNN30 measure could. The increased pNN50 count in the CM session group argues for an increased complexity in the dynamics of the heart after the practice – while there is a significant increase of parasympathetic activity, as evidenced by the increase in the HF component and indicative of a calming effect, the variation in the inter-RR intervals has increased. The results relating to the pNN30 statistic bear this out – the significantly larger pNN30 count in the meditation group implies a more complex signal after the practice as compared to SR. The RMSSD is known to be correlated to the pNN50[16] and the significant increase in the RMSSD after the CM session is indicative of this fact.

**Figure 1:**
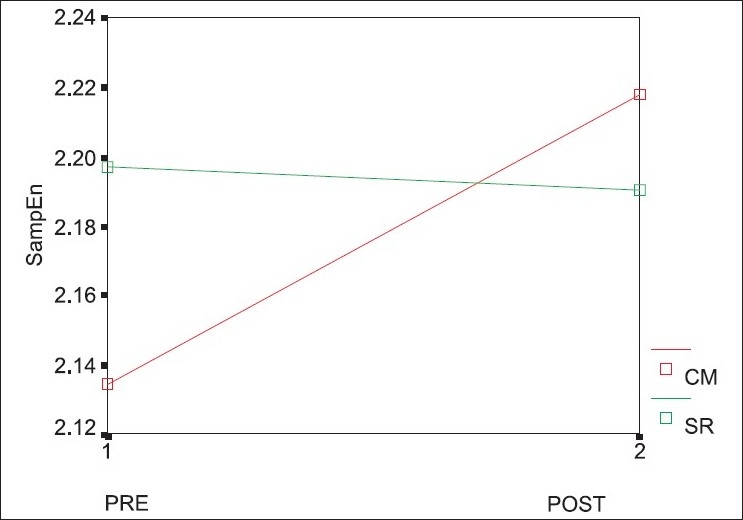
Sampling entropy

Both sampling and approximate entropy measure the complexity in the dynamics of a time series. SampEn has an advantage over approximate entropy, its precursor, in having better consistency.[10] SampEn depends on two parameters, *m* and *r*, *m* being the length of the sequence being matched and *r* being the tolerance limit. The SampEn calculations were performed in this study with *m*=2 and *r*=0.2 because this is suited to a short time-series (300 RR-intervals in this case). An increased value of SampEn is indicative of increased complexity in the signal. There is a multitude of evidence to support a deterioration of complexity (decrease in entropy) in different kinds of time series in subjects with ill-health.[17] In the context of heart-rate variability, a decrease in entropy has been associated with an increased risk of cardiac failure. Given the subjective feeling of relaxation that is experienced by individuals after the practice of CM, an increase in the SampEn was hypothesized in the period immediately after the practice. The accompanying graph [Figure 1] shows the changes that occurred following the two practices. The SampEn increased in the CM group and decreased marginally in the other group. While the increase in the CM group is not significant (*P*=0.268), a comparison of the effect sizes for each practice is instructive. The effect size gives an absolute measure of the change seen and is defined to be the difference in means divided by the standard deviation of the difference scores. The effect size is 0.2 for the CM group and only 0.02 for the control group. A *post hoc* calculation of the power showed that the PRE-POST study had a power of only 0.3. This suggests that a significant difference could be found by increasing the sample size (which increases the power) or altering the design. Another improvement in the current study would be to use an instrument with a faster sampling rate than the one used here (256 Hz). An improved version of this study, focusing on the SampEn, will be reported later.

The difference in heart rate variability between men and women has been commented on before,[2] where it was observed that baroreflex responsiveness is attenuated and vagal activity is augmented in women compared with men. Because the LF component of the heart rate variability reflects, in part, the baroreflex-mediated control of the heart rate, women tend to display a lower value of power in the LF spectrum of the heart rate variations. The absolute powers displayed in Table 1 demonstrate this. Thus, the values in the LF and HF columns in Table 1 would be interchanged for men.

## CONCLUSIONS

This study has shown that the state of relaxation after CM results in parasympathetic dominance in women, as evidenced by the increased HF component of the RR-interval series. The fact that the dynamics of heart rate variability is different in women as compared to men has also been seen. The observed significant increase in the pNNx counts and the increase in the SampEn after the practice when the subjects report a sense of calmness and well-being would seem to imply a complex dynamics that requires further study.
